# Medicare Advantage Benefits Design and Access to Cardiovascular Care

**DOI:** 10.1001/jamanetworkopen.2026.5439

**Published:** 2026-04-07

**Authors:** Jessica I. Billig, Shining Yang, Jennifer R. Cardin, Joseph H. Joo, Changchuan Jiang, Joshua M. Liao

**Affiliations:** 1 Department of Plastic Surgery, University of Texas Southwestern Medical Center, Dallas; 2 Division of General Internal Medicine, Department of Internal Medicine, University of Texas Southwestern Medical Center, Dallas; 3 Division of Hematology and Oncology, Department of Internal Medicine, University of Texas Southwestern Medical Center, Dallas; 4 Peter O’Donnell Jr School of Public Health, University of Texas Southwestern Medical Center, Dallas

## Abstract

**Importance:**

Financial and geographic barriers are associated with worse cardiovascular outcomes, underscoring the need to improve access to cardiovascular care. Medicare Advantage (MA) plans offer reduced cost-sharing for cardiologists, potentially mitigating financial barriers to cardiovascular care through minimizing out-of-pocket costs.

**Objectives:**

To determine how availability of Medicare Advantage plans with reduced cost-sharing for cardiologists has changed over time, whether plans offer reduced cost-sharing to beneficiaries in communities with more or less cardiologist supply or cardiovascular care infrastructure, and whether such benefits are offered by high-quality plans.

**Design, Setting, and Participants:**

This cross-sectional study of MA benefit design related to cardiovascular care used plan benefits package and MA enrollment data from December 31, 2022, to December 31, 2024. Participants were MA beneficiaries. Data were analyzed from March 1 to May 23, 2025.

**Exposures:**

MA plans with reduced cost-sharing for cardiologists.

**Main Outcomes and Measures:**

The primary outcomes were (1) enrollment patterns in MA plans offering reduced-cost sharing for cardiologists (measured as quarterly enrollment in such plans from 2022 to 2024), (2) number of MA plans with reduced cost-sharing for cardiologists mapped to county-level cardiologist supply (measured as the number of cardiologists per 1000 MA beneficiaries) and cardiovascular care infrastructure (measured via individual measures and a composite index of 3 county-level measures), and (3) quality of these plans vs other MA plans (measured via 3 metrics of cardiovascular care–related quality from the Healthcare Effectiveness Data and Information Set).

**Results:**

Among 2993 plans with reduced cost-sharing for cardiologists and 3143 counties, the number of plans offering reduced cost-sharing for cardiologists (from 134 of 6448 [2.1%] in 2022 to 158 of 6808 [2.3%] in 2024) and enrollment in such plans (1.4 million in 2022 to 1.5 million in 2024) increased during the study period. Across counties, the number of MA plans with reduced cost-sharing for cardiologists varied considerably compared with cardiologist supply and cardiovascular care infrastructure. Reduced cost-sharing plans had greater care quality compared with other MA plans (116 of 158 [73.4%] with reduced cost-sharing for cardiologists being high quality vs 2508 of 6650 [37.7%]; *P* < .001).

**Conclusions and Relevance:**

In this cross-sectional study of plans with reduced cost-sharing for cardiologists, the number of plans increased over time and tended to be higher quality compared with other MA plans. However, persistent geographic variability in number of plans offered, cardiologist supply, and cardiovascular care infrastructure underscore the need for additional strategies to minimize financial and geographic barriers for cardiovascular care.

## Key Points

**Questions:**

Has the availability of Medicare Advantage (MA) plans with reduced cost-sharing for cardiologists changed over time, are plans offered to beneficiaries in communities with more or less cardiologist supply or cardiovascular care infrastructure, and are such benefits offered by high-quality plans?

**Findings:**

In this cross-sectional study of 2993 plans with reduced cost-sharing for cardiologists and 3143 counties, the number of MA plans with reduced cost-sharing for cardiologists varied considerably compared with cardiologist supply and cardiovascular care infrastructure. Reduced cost-sharing plans had greater care quality compared with other MA plans.

**Meaning:**

Persistent geographic variability in the number of plans offered, cardiologist supply, and cardiovascular care infrastructure underscores the need for additional strategies to minimize financial and geographic barriers for cardiovascular care.

## Teaser

This cross-sectional study assesses how availability of Medicare Advantage plans with reduced cost-sharing for cardiologists has changed over time, whether plans offered reduced cost-sharing to beneficiaries in communities with more or less cardiologist supply or cardiovascular care infrastructure, and whether such benefits were offered by high-quality plans.

## Introduction

Heart disease is common among older adults—with approximately 42% of Medicare beneficiaries affected by at least 1 heart condition^1^—and has been the leading cause of death in the US for the last 100 years.^2^ Financial and geographic barriers to accessing cardiovascular care are among several factors that contribute to heart disease–related mortality. Some barriers to accessing care include high out-of-pocket (OOP) expenses, lack of insurance or underinsurance, limited cardiologist supply, and limited cardiovascular care infrastructure (eg, hospitals with cardiac rehabilitation facilities, adult cardiac services, and/or cardiac intensive care units [ICUs]).^3,4,5,6,7,8,9,10,11^

In particular, high OOP expenses have been associated with worse cardiovascular medication adherence,^12,13,14,15,16^ delays in seeking medical care,^17,18^ and in turn, increased cardiovascular morbidity and mortality.^19,20,21,22,23^ OOP expenses have increased over time, with patients bearing more of their health care expenses, thereby exacerbating known access challenges.^5,24,25,26^ One potential way to address the financial barriers and improve patient outcomes is through decreasing OOP expenses (ie, reduction in cost-sharing). Medicare Advantage (MA) plans, now the predominant insurer of older adults nationwide, offer ancillary benefits including reduced cost-sharing—that is, lower copayments, coinsurance, or deductibles—for cardiologists. Such offerings might attract beneficiaries and potentially mitigate their financial barriers to seeking cardiovascular care. However, little is known about these plans; in particular, it is unknown how availability to MA plans with reduced cost-sharing for cardiologists has changed over time, whether plans offer reduced cost-sharing to beneficiaries in communities with more or less cardiologist supply or cardiovascular care infrastructure, and whether such benefits are offered by high-quality plans.

## Methods

### Data Sources

We used the plan benefits package and MA enrollment data from December 31, 2022, to December 31, 2024, to perform a cross-sectional study of MA plan benefit design related to cardiovascular care, following the Strengthening the Reporting of Observational Studies in Epidemiology (STROBE) reporting guideline for cross-sectional studies. The Institutional Review Board of the University of Texas Southwestern Medical Center deemed this study as non–human participant research that did not require approval or informed consent. We restricted our analysis to plans that were widely available to the public (ie, health maintenance organizations [HMOs] and preferred provider organizations [PPOs]). These databases contain the number of MA plans, enrollment, plan type (HMO and PPO), and plan attributes (reduced cost-sharing for specialists, including cardiologists).

We extracted cardiologist supply and cardiovascular care infrastructure from the 2023 Area Health Resource Files of the Health Resources and Services Administration, which are the most recent data available, and 2022 to 2024 MA contract-level quality cardiac-related care measures from the Healthcare Effectiveness Data and Information Set (HEDIS). Cardiologists were considered medical doctors only, precluding advanced practice professionals, as defined by self-designated practice specialties to the American Medical Association, whose physician master file dataset was sourced for the Health Resources and Service Administration for their Area Health Resource Files dataset.

### Study Cohort and Measures

MA plans with reduced cost-sharing for cardiologists were defined as plans offering at least 1 of the following for cardiologist visits: reduced coinsurance, reduced deductible, or reduced copayment. This is a supplemental benefit available in specific MA plans with these specific variables. In our analytic sample, qualifying plans provided either reduced coinsurance or reduced copayments; there were no plans that offered reduced deductibles. We assessed the total number of plans with reduced cost-sharing for cardiologists and quarterly enrollment in such plans from 2022 to 2024. We defined MA plans with reduced cost-sharing for other medical specialties as plans that offer reduced cost-sharing for any of the 9 medical subspecialty services: geriatrics, allergy, endocrinology, gastroenterology, infectious disease, nephrology, oncology (medical or surgical), pulmonology, and rheumatology. These plans served as a comparison group to evaluate how cost-sharing reduction has changed over time regardless of specialty. We then compared the total number of MA plans and enrollment in those with reduced cost-sharing for cardiologists with all MA plans and all MA plans with reduced cost-sharing for other medical subspecialty services.

We mapped 2023 county-level MA plans with reduced cost-sharing for cardiologists, as well as county-level distribution of cardiologist supply and cardiovascular care infrastructure. In the map for MA plans, we excluded plans with fewer than 10 enrolled beneficiaries at the county level (72 of 144), as Medicare does not publish these data due to the Health Insurance Portability and Accountability Act. Because of the skewed nature of the MA plan distribution, counties with 0 plans and counties falling within the top 1% of plan counts were assigned to distinct categories, with the remaining counties stratified into tertiles.

The county-level geographic distribution of cardiologist supply was calculated as the number of cardiologists per 1000 MA beneficiaries. Of 3143 counties, 35 lacked data on the number of MA enrollees, precluding us from calculating cardiologists per 1000 MA enrollees (labeled as missing MA beneficiary data). Because of the skewed nature of the cardiologist supply, counties with 0 cardiologists and counties falling within the top 1% of density of cardiologists to beneficiaries were assigned to distinct categories, with the remaining counties stratified into quartiles. Geographic variation in cardiovascular care infrastructure was assessed both through individual measures (ie, the number of cardiac rehabilitation facilities, the number of hospitals with adult cardiac services, and the number of hospitals with cardiac ICUs) and using a composite index of 3 county-level measures (eMethods 1 and eTable 1 in Supplement 1). To assess overlap among the infrastructure components prior to index construction, we examined a pairwise correlation matrix across the 3 measures. Correlations were uniformly low (all *r* ≤ 0.65) (eFigure 1 in Supplement 1), suggesting limited redundancy; therefore, we retained all measures in the composite index. The composite score ranged from 0 to 3.0, with each element receiving a value of 0 if it was not present in the county and 1.0 if it was present (eMethods 2 in Supplement 1). We then stratified the counties into geographic regions (Northeast, South, Midwest, and West).

Finally, using data from 2022 to 2024, we assessed plan quality based on 3 cardiovascular care–related quality HEDIS metrics, as well as a cardiovascular care quality index that combined the 3 individual metrics. These 3 metrics were statin therapy adherence, cardiac rehabilitation attendance, and adequately controlled high blood pressure. To create the cardiovascular care quality index, we first assigned each plan point based on its performance on each underlying measure: for each quality measure, plans in the first (lowest-quality) quartile received 0 points, those in the second or third quartile received 0.5 points, and those in the fourth (highest-quality) quartile receive 1.0 point. We then summed the plan points across the 3 measures into a composite score defining plans with composite scores less than 1.0 as low quality, plans with scores from 1.0 to less than 2.5 as moderate quality, and plans with scores 2.5 or greater as high quality. We also looked at each HEDIS measure individually (eTable 2 in Supplement 1). We dropped MA contracts that were missing all 3 HEDIS measures (20 of 568 in 2022; 27 of 615 in 2023; and 32 of 621 in 2024). We then compared plan quality between MA plans offering reduced cost-sharing for cardiologists vs those that did not, imputing missing values using multiple imputation by chained equations.^27^

### Statistical Analysis

Data were analyzed using counts, percentages, and ratios (ie, number of cardiologists per 1000 MA beneficiaries). Categorical variables were compared using χ^2^ tests. Two-sided *P* < .05 indicated statistical significance. We used Python, version 3.12.3 (Python Software Foundation) to perform statistical analyses and QGIS Desktop, version 3.34.15 (QGIS), to generate maps. All data were analyzed from March 1 to May 23, 2025.

For sensitivity analyses, we evaluated the robustness of the cardiovascular care–related HEDIS quality index to alternative index construction approaches. First, we varied the scoring algorithm used for each component measure. Instead of categorizing each measure into quartiles and assigning a score of 0 for the lowest quartile, 0.5 for the middle 2 quartiles, and 1.0 for the highest quartile, we categorized each measure into tertiles and assigned scores of 0 to the first tertile, 0.5 to the second tertile, and 1.0 to the third tertile (eTable 3 in Supplement 1). Second, because the cardiac rehabilitation component exhibited substantial geographic sparsity (many counties with no cardiac rehabilitation facility), we reconstructed the quality index excluding the cardiac rehabilitation measure and re-estimated all analyses using the remaining 2 measures (eTable 4 in Supplement 1). Additionally, to assess whether observed improvements in the cardiovascular care quality index were due to secular or systematic changes in HEDIS reporting and performance rather than cardiovascular care specifically, we conducted a negative-control analysis using 3 non–cardiovascular-related HEDIS measures: adults’ access to preventive and/or ambulatory services, kidney health evaluation for patients with diabetes, and transitions of care (eTable 5 in Supplement 1).

## Results

We included 2993 plans with reduced cost-sharing for cardiologists and 3143 counties in the analysis. From 2022 to 2024, there were increases in both the number of and beneficiary enrollment in all MA plans and MA plans with reduced cost-sharing for cardiologists (Table 1). The number of all MA plans available increased from 6448 in 2022 to 6808 in 2024, with enrollment growing from 29.5 to 33.9 million beneficiaries. During this time, PPO plans increased from 2307 (35.8%) to 2670 (39.2%). MA plans with reduced cost-sharing for cardiologists also increased from 134 plans in 2022 to 158 plans in 2024, with enrollment increasing from 1.4 million to 1.5 million during the same period. However, the proportion of MA plans with reduced cost-sharing for cardiologists remained stable (2.1% in 2022 to 2.3% in 2024). Among these plans, there was a shift from HMO to PPO, with PPO increasing from 46 of 134 (34.3%) in 2022 to 78 of 158 (49.4%) in 2024.

**Table 1.** MA Plans and Reduced Cost-Sharing Plans for Cardiology and Medical Subspecialties, 2022 to 2024

Plan^a^	No. (%) of plans		
	2022	2023	2024
**All MA plans**			
Overall	6448 (100)	6913 (100)	6808 (100)
HMO	4141 (64.2)	4305 (62.3)	4138 (60.8)
PPO	2307 (35.8)	2608 (37.7)	2670 (39.2)
Total enrollment	29 528 918	31 997 748	33 948 259
**MA reduced cost-sharing plans for cardiology**			
Overall	134 (2.1)	144 (2.1)	158 (2.3)
HMO	88 (65.7)	90 (62.5)	80 (50.6)
Reduced coinsurance	52 (38.8)	52 (36.1)	53 (33.5)
Reduced copayment	36 (26.9)	38 (26.4)	27 (17.1)
PPO	46 (34.3)	54 (37.5)	78 (49.4)
Reduced coinsurance	34 (25.4)	42 (29.2)	71 (44.9)
Reduced copayment	12 (9.0)	12 (8.3)	7 (4.4)
Total enrollment	1 397 640	1 496 397	1 520 662
**MA reduced cost-sharing plans for medical subspecialties^b^**			
Overall	131 (2.0)	145 (2.1)	163 (2.4)
HMO	88 (67.2)	90 (62.1)	85 (52.1)
Reduced coinsurance	52 (39.7)	52 (35.9)	53 (32.5)
Reduced copayment	36 (27.5)	38 (26.2)	32 (19.6)
PPO	43 (32.8)	55 (37.9)	78 (47.9)
Reduced coinsurance	34 (26.0)	42 (29.0)	71 (43.6)
Reduced copayment	9 (6.9)	13 (9.0)	7 (4.3)
Total enrollment	1 404 600	1 500 973	1 562 689

Abbreviations: HMO, health maintenance organization; MA, Medicare Advantage; PPO, preferred provider organization.

^a^ Reduced cost-sharing is through reduced coinsurance or copayment.

^b^ Include geriatrics, allergy, endocrinology, gastroenterology, infectious disease, nephrology, medical or surgical oncology, pulmonology, and rheumatology.

Similar patterns were observed for MA plans offering reduced cost-sharing for other medical subspecialists, with these plans increasing from 131 plans with 1.4 million beneficiaries in 2022 to a total of 163 plans with 1.6 million beneficiaries in 2024, and PPO plans increasing from 43 of 131 (32.8%) to 78 of 163 (47.9%). This highlights an increase both in the number of and beneficiary enrollment in MA plans that offer reduced cost-sharing regardless of subspecialty.

**Figure 1.**
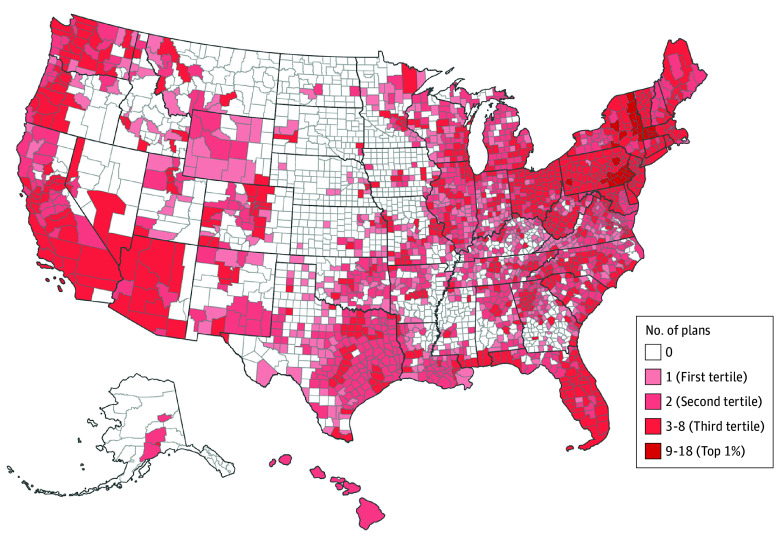
Heat Map of Geographic Variation in Medicare Advantage (MA) Plans With Reduced Cost-Sharing for Cardiologists, 2023 Data are representative of cardiologists with valid enrollment (ie, more than 10 enrolled MA beneficiaries at a county level).

The 2023 county-level distribution of MA plans with reduced cost-sharing for cardiologists was heterogeneous (Figure 1). Overall, 1118 of 3143 counties across the US (35.6%) had no MA plans with reduced cost-sharing for cardiologists. Many of these counties were located in Montana and parts of the Midwest, including Kansas, Iowa, Nebraska, North Dakota, and South Dakota. Areas in the third highest tertile of number of MA plans with reduced cost-sharing for cardiologists were located in parts of the West, including Arizona, California, and Washington and all states in the Northeast region of the US. Florida also had a higher number of plans with reduced cost-sharing for cardiologists. Counties with the highest number of MA plans with reduced cost-sharing for cardiologists (top 1%) were located in Massachusetts, New York, and Pennsylvania.

**Figure 2.**
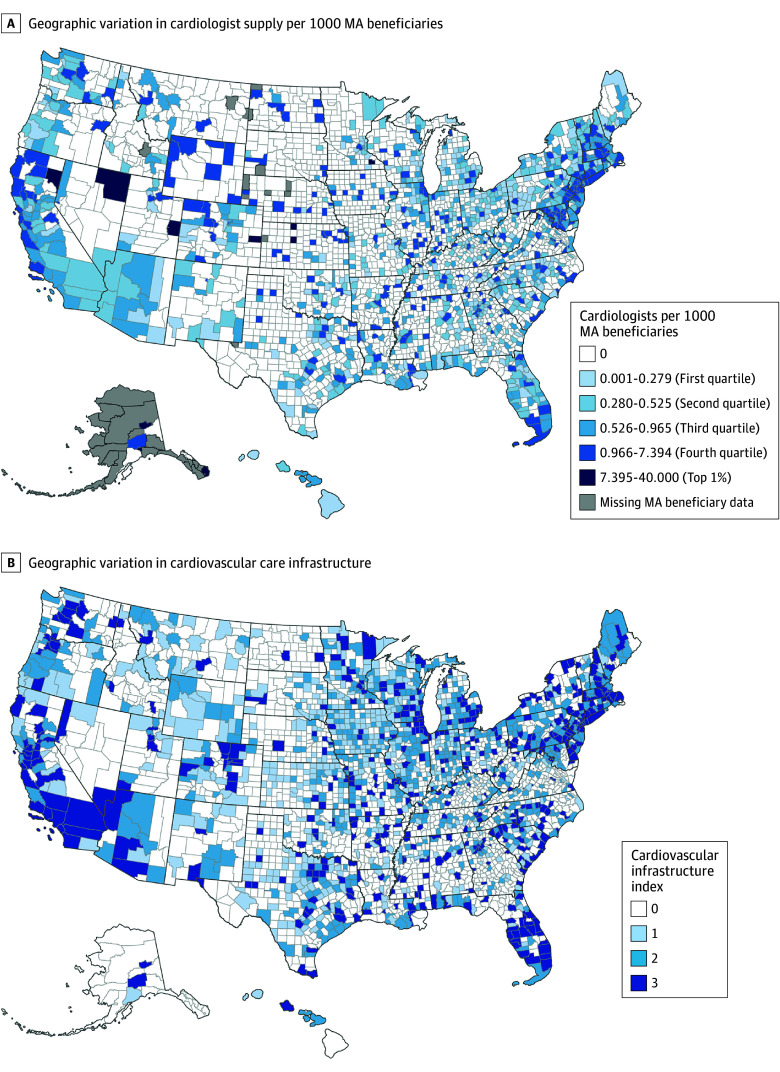
Heat Maps of Geographic Variation in County-Level Cardiologist Supply Per 1000 Medicare Advantage (MA) Beneficiaries and Cardiovascular Infrastructure, 2023 A cardiovascular infrastructure index of 0 indicates counties with no cardiovascular care infrastructure; an index of 3 indicates counties with the highest level of cardiovascular care infrastructure.

Cardiologist supply varied geographically across the US (Figure 2A). There were no cardiologists in 1953 of 3143 counties (62.1%), and a substantial number of these were located in the Midwest in Iowa, Kansas, Minnesota, Missouri, Nebraska, North Dakota, and South Dakota, as well as the West in Idaho, Montana, and Utah. The top 1% of counties with the highest cardiologist supply were located in Anchorage, Alaska; Charlottesville, Virginia; Cheyenne, Colorado; Elko, Nevada; Ellis, Kansas; Fairbanks North Star, Alaska; Grand, Utah; Ketchikan Gateway, Alaska; Lassen, California; Olmsted, Minnesota; Phillips, Kansas; and Suffolk, Massachusetts. Many counties with the highest ratios of cardiologists per MA beneficiary likely had low MA enrollment (ie, Cheyenne County, Colorado; Ketchikan Gateway Borough, Alaska). Conversely, many densely populated counties fell into the bottom tertile due to lower ratios resulting from a higher number of MA beneficiaries (ie, Livingston County, Michigan; Pinal County, Arizona).

More than half (1586 of 3143 [50.5%]) of counties in the US had no existing cardiovascular care infrastructure, with Nebraska, Nevada, North Dakota, and South Dakota being among the states with the most counties with an index of 0 for cardiovascular care infrastructure (Figure 2B). More than 450 counties had a cardiovascular care infrastructure index of 3.0 (highest index). These counties were in the West in California, Oregon, and Washington; the Northeast in Massachusetts, New Jersey, New York, and Pennsylvania; and the South in Florida. The percentage of counties with individual cardiovascular care infrastructure components varied, with 1418 of 3143 (45.1%) offering cardiac rehabilitation facilities, 1125 of 3143 (35.8%) offering adult cardiac services, and 525 of 3143 (16.7%) offering availability of cardiac ICUs (eFigures 2-4 in Supplement 1). The overlap between cardiologist supply and cardiovascular care infrastructure is not exact. However, in higher populated counties, there was higher cardiologist supply (Figure 2A) and cardiovascular care infrastructure (Figure 2B).

Approximately one-third of counties (1122 of 3143 [35.7%]) had 0 enrollment in MA plans with reduced cost-sharing for cardiologists. Within these counties, 1048 (93.4%) had no cardiologists and 800 (71.3%) had no cardiovascular care infrastructure. Moreover, 1084 (96.6%) of these counties had both 0 cardiologists and no cardiovascular care infrastructure. There were few counties that had MA plans with reduced cost-sharing for cardiologists, cardiologist supply, and cardiovascular care infrastructure. In some counties, there were differences in the distribution of cardiologist supply and cardiovascular care infrastructure. For example, Cowley County, Kansas, and Dodge County, Wisconsin, had a cardiologist supply of 0 but an index of 3.0 for cardiovascular care infrastructure (highest index).

Over time, MA plans with reduced cost-sharing for cardiologists increasingly comprised high-quality plans, in contrast to other MA plans (Table 2). Compared with other MA plans, the percentage of high-quality MA plans with reduced cost-sharing for cardiologists was slightly lower in 2022 (MA plans with reduced cost-sharing for cardiologists: 9 of 134 [6.7%]; other MA plans: 571 of 6314 [9.0%]; *P* = .44), but substantially higher by 2024 (MA plans with reduced cost-sharing for cardiologists, 116 of 158 [73.4%]; other MA plans, 2508 of 6650 [37.7%]; *P* < .001). To assess whether any change in plan quality over time was primarily driven by any single HEDIS component, we looked at change over time of each individual HEDIS measure (eTable 2 in Supplement 1). This shift toward high-quality plans offering reduced cost-sharing for cardiology was seen in for all 3 HEDIS measures separately, with statin adherence increasing from 33 of 134 (24.6%) to 85 of 158 (53.8%), cardiac rehabilitation increasing from 47 of 134 (35.1%) to 117 of 158 (74.1%), and controlling blood pressure increasing from 24 of 134 (17.9%) to 105 of 158 (66.5%) by 2024.

**Table 2.** Quality of Care: Medicare Advantage Reduced Cost-Sharing Plans for Cardiologists vs Other Subspecialties, 2022 to 2024^a^

Quality of plan	2022			2023			2024		
	Cardiology	Other subspecialty	*P* value^b^	Cardiology	Other subspecialty	*P* value^b^	Cardiology	Other subspecialty	*P* value^b^
Overall	134 (100)	6314 (100)	NA	144 (100)	6769 (100)	NA	158 (100)	6650 (100)	NA
Low (0, 0.5, and 1.0)	35 (26.1)	1927 (30.5)	.44	15 (10.4)	1500 (22.2)	<.001	4 (2.5)	764 (11.5)	<.001
Moderate (1.5, 2.0)	88 (65.7)	3313 (52.5)		51 (35.4)	2954 (43.6)		36 (22.8)	2977 (44.8)	
High (2.5, 3.0)	9 (6.7)	571 (9.0)		78 (54.2)	1845 (27.3)		116 (73.4)	2508 (37.7)	
Unknown	2 (1.5)	503 (8.0)	NA	0	470 (6.9)	NA	2 (1.3)	401 (6.0)	NA

Abbreviation: NA, not applicable.

^a^ Data are presented as the No. (%) of plans. Other subspecialties include geriatrics, allergy, endocrinology, gastroenterology, infectious disease, nephrology, medical or surgical oncology, pulmonology, and rheumatology. Healthcare Effectiveness Data and Information Set metrics for quality of care included statin therapy for patients with cardiovascular disease, cardiac rehabilitation, and controlling high blood pressure.

^b^ Obtained by comparing high-quality vs low- and moderate-quality plans.

### Sensitivity Analyses

Our findings were robust to alternative specifications of the cardiovascular care–related HEDIS quality index. Reconstructing the index using tertile-based scoring (0, 0.5, and 1.0 across tertiles) yielded results consistent with the primary quartile-based scoring approach (eTable 3 in Supplement 1). Similarly, excluding the cardiac rehabilitation component and constructing a 2-measure index did not materially change the direction of associations observed in main analyses (eTable 4 in Supplement 1).

In the negative-control analysis (eTable 5 in Supplement 1), the percentage of high-quality plans defined by the 3 non–cardiovascular-related HEDIS measures did not uniformly increase during the study period: the percentage of high-quality MA plans with reduced cost-sharing for cardiologists increased in the kidney health evaluation for patients with diabetes measure over time, but not in the other 2 measures. This lack of consistent improvement across unrelated HEDIS measures suggests that the observed increase in the cardiovascular care quality index is unlikely to be driven solely by broad, system-wide changes in HEDIS reporting or performance. Overall, these sensitivity analyses were consistent with findings from our main analyses.

## Discussion

In this study of MA benefits design for cardiovascular care, the number of and enrollment in MA plans with reduced cost-sharing for cardiologists increased over time. There was substantial variability in enrollment in MA plans with reduced cost-sharing for cardiologists as well as county-level cardiologist supply and infrastructure, while MA plans with reduced cost-sharing for cardiologists also tended to have higher cardiovascular care quality compared with other MA plans.

Our study mirrors other findings on increased enrollment in MA plans offering expanded supplemental benefits. Previous studies^28,29,30^ have found that an increase in MA plans offering ancillary benefits targeting long-term care services have been shown to improve care quality. The benefit design of other MA plans has expanded to help chronically ill patients such as those with cardiovascular disease to maintain health through medical and nonmedical benefits.^31,32,33^ Our analysis generated directionally similar findings, demonstrating an increase in the number of and enrollment in MA plans with reduced cost-sharing for cardiologists. However, on an absolute scale, the uptake of these plans with ancillary benefits remains low among Medicare beneficiaries, suggesting that other factors beyond these benefits alone may drive plan selection.^34,35^ Nonetheless, these ancillary MA benefits have the potential to address social and financial barriers to provide better access to care.

We found substantial geographic variability in overall plans with reduced cost-sharing for cardiologists compared with cardiologist supply and cardiovascular care infrastructure. Specifically, most of the counties with no MA plans with reduced cost-sharing for cardiologists also had limited cardiologist supply and infrastructure. Numerous studies have shown that greater access to cardiologists improves cardiovascular outcomes.^19,20,21,22^ However, rural and underserved communities often lack access to sufficient cardiologists and cardiovascular care infrastructure, with as many as 62% of US counties lacking a cardiologist and nearly 50% of cardiologists practicing in areas servicing 25% of Medicare beneficiaries.^3,4,9^ Furthermore, Medicare beneficiaries in areas with fewer cardiologists have higher rates of 30-day mortality.^23^ Similar to cardiologist growth favoring urban areas, cardiac ICUs are increasingly found in urban areas, which is corroborated by our results.^36^ Last, geographic variation in cardiac rehabilitation access is of particular concern given its underutilization and the known benefits for older adults.^37^ However, solutions aimed at addressing a lack of infrastructure (eg, building more cardiac ICUs or rehabilitation centers) are challenging to implement, and while there has been an increase in the number of cardiologists being trained, they are largely recruited to urban centers that may not address Medicare beneficiaries’ cardiovascular needs.^8^ Given these challenges, expanding MA plan benefit design and improving access to plans that minimize the financial barriers for cardiovascular care may be one avenue to improve quality and outcomes of older adults.

Our findings about plan quality and reduced cost-sharing for cardiologists highlight the potential to improve population outcomes for patients by aligning lower OOP expenses with better quality. Our findings build from prior studies demonstrating that reducing OOP costs improves adherence and access to preventive cardiovascular care.^19,38^ An improvement in access to cardiovascular care is particularly salient for cardiovascular rehabilitation, which historically has a very low uptake among Medicare beneficiaries, despite being a cost-effective way of reducing mortality, decreasing hospitalization, and increasing functional capacity and quality of life.^17,39,40^ Therefore, addressing the financial barriers to seeking cardiovascular care through plans that reduce cost-sharing for cardiologists can be one avenue to improve access to care.

### Limitations

Our study has limitations. Its cross-sectional design precludes inference about causality. Dataset limitations also preclude our ability to quantify the extent of cost-sharing reductions as well as other plan features (eg, sufficiency of provider networks, cost of insurance premiums). The lack of data on patient-level utilization also creates the need for future work assessing the impact of cost-sharing reductions on patient outcomes.

## Conclusions

In this cross-sectional study of MA plan benefit design, plans with reduced cost-sharing for cardiologists increased over time and tended to be of higher quality compared with other MA plans. However, persistent geographic variability in plan enrollment, cardiologist supply, and cardiovascular care infrastructure underscore the need for additional strategies to minimize financial and geographic barriers for cardiovascular care.

## Notes

Supplement 1. **eMethods 1.** Construction of the County-Level Cardiovascular Care Infrastructure Index **eTable 1.** Univariate Distribution of County-Level Cardiovascular Care Infrastructure **eFigure 1.** Correlation Matrix of County-Level Cardiovascular Infrastructure Components **eMethods 2.** Construction of the Effectiveness-of-Care Index Based on HEDIS Measures **eTable 2.** Component-Wise Breakdown of Cardiac HEDIS Measures Within Medicare Advantage Reduced Cost-Sharing Plans for Cardiologists vs Other, 2022-2024 **eTable 3.** Quality of Care: Medicare Advantage Reduced Cost-Sharing Plans for Cardiologists vs Other, 2022-2024 **eTable 4.** Quality of Care: Medicare Advantage Reduced Cost-Sharing Plans for Cardiologists vs Other, 2022-2024 **eTable 5.** Component-Wise Breakdown of Non–Cardiac-Related HEDIS Measures Within Medicare Advantage Reduced Cost-Sharing Plans for Cardiologists vs Other, 2022-2024 **eFigure 2.** Geographic Variation in County-Level Cardiovascular Rehabilitation Facilities, 2023 **eFigure 3.** Geographic Variation in County-Level Adult Cardiology Services, 2023 **eFigure 4.** Geographic Variation in County-Level Cardiovascular ICUs, 2023

Supplement 2.
Data Sharing Statement
