# Supplementary material for: Medicare Advantage Benefits Design and Access to Cardiovascular Care
Source: JAMA Netw Open. 2026 Apr 7;9(4):e265439. doi: 10.1001/jamanetworkopen.2026.5439 (PMC13058761; doi:10.1001/jamanetworkopen.2026.5439)
Supplement: Supplement 1. — eMethods 1. Construction of the County-Level Cardiovascular Care Infrastructure Index eTable 1. Univariate Distribution of County-Level Cardiovascular Care Infrastructure eFigure 1. Correlation Matrix of County-Level Cardiovascular Infrastructure Components eMethods 2. Construction of the Effectiveness-of-Care Index Based on HEDIS Measures eTable 2. Component-Wise Breakdown of Cardiac HEDIS Measures Within Medicare Advantage Reduced Cost-Sharing Plans for Cardiologists vs Other, 2022-2024 eTable 3. Quality of Care: Medicare Advantage Reduced Cost-Sharing Plans for Cardiologists vs Other, 2022-2024 eTable 4. Quality of Care: Medicare Advantage Reduced Cost-Sharing Plans for Cardiologists vs Other, 2022-2024 eTable 5. Component-Wise Breakdown of Non–Cardiac-Related HEDIS Measures Within Medicare Advantage Reduced Cost-Sharing Plans for Cardiologists vs Other, 2022-2024 eFigure 2. Geographic Variation in County-Level Cardiovascular Rehabilitation Facilities, 2023 eFigure 3. Geographic Variation in County-Level Adult Cardiology Services, 2023 eFigure 4. Geographic Variation in County-Level Cardiovascular ICUs, 2023 [file jamanetwopen-e265439-s001.pdf]

Supplementary Online Content

Billig JI, Yang S, Cardin J, Joo JH, Jiang C, Liao JM. Medicare Advantage benefits design and access to cardiovascular care. *JAMA Netw Open.* 2026;9(4):e265439. doi:10.1001/jamanetworkopen.2026.5439

eMethods 1. Construction of the County-Level Cardiovascular Care Infrastructure Index
eTable 1. Univariate Distribution of County-Level Cardiovascular Care Infrastructure
eFigure 1. Correlation Matrix of County-Level Cardiovascular Infrastructure Components
eMethods 2. Construction of the Effectiveness-of-Care Index Based on HEDIS Measures
eTable 2. Component-Wise Breakdown of Cardiac HEDIS Measures Within Medicare Advantage Reduced Cost-Sharing Plans for Cardiologists vs Other, 2022-2024
eTable 3. Quality of Care: Medicare Advantage Reduced Cost-Sharing Plans for Cardiologists vs Other, 2022-2024
eTable 4. Quality of Care: Medicare Advantage Reduced Cost-Sharing Plans for Cardiologists vs Other, 2022-2024
eTable 5. Component-Wise Breakdown of Non–Cardiac-Related HEDIS Measures Within Medicare Advantage Reduced Cost-Sharing Plans for Cardiologists vs Other, 2022-2024
eFigure 2. Geographic Variation in County-Level Cardiovascular Rehabilitation Facilities, 2023
eFigure 3. Geographic Variation in County-Level Adult Cardiology Services, 2023
eFigure 4. Geographic Variation in County-Level Cardiovascular ICUs, 2023

This supplementary material has been provided by the authors to give readers additional information about their work.

© 2026 Billig JI et al. *JAMA Network Open*.

eMethods 1. Construction of the County-Level Cardiovascular Care Infrastructure Index

We selected three cardiovascular care infrastructures from the 2023-2024 county-level Area Health Resources File: number of hospitals with cardiac intensive care units, number of hospitals with adult cardiac services, and number of hospitals with cardiac rehabilitations. Table shows the univariate distribution of each variable.

eTable 1. Univariate Distribution of County-Level Cardiovascular Care Infrastructure

	**Hospitals with Cardiac Rehabilitations**	**Hospitals with Adult Cardiac Services**	**Hospitals with Cardiac Intensive Care Units**
Number of Total Counties	3,143	3,143	3,143
Mean	0.69	0.67	0.29
Std Dev	1.22	1.68	1.00
Min	0	0	0
25%	0	0	0
50%	0	0	0
75%	1	1	0
Max	23	42	27

Due to the skewed distribution of the data, we converted each variable into a binary indicator (0 or 1) representing the presence or absence of the respective infrastructure in each county. We then constructed a composite index ranging from 0 to 3 by summing these 3 binary indicators at the county-level.

© 2026 Billig JI et al. *JAMA Network Open*.

eFigure 1. Correlation Matrix of County-Level Cardiovascular Infrastructure Components

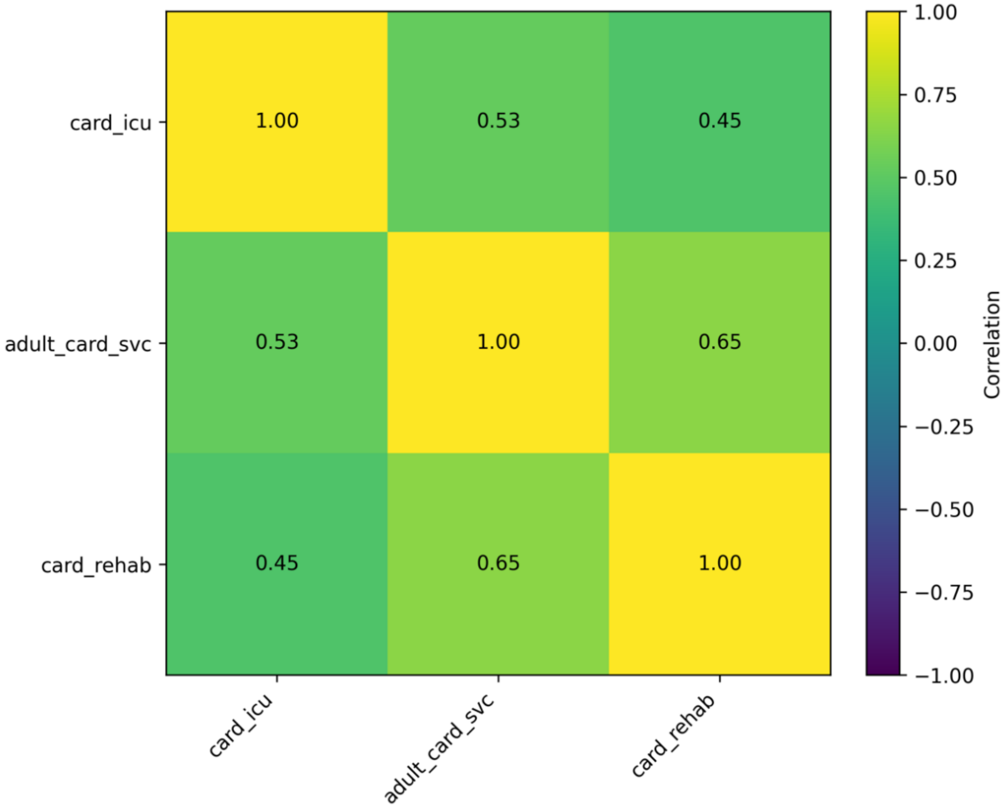

This figure shows pairwise *Pearson* correlation coefficients (r) among the county-level cardiovascular infrastructure variables (hospitals with cardiac ICU, cardiac rehabilitation, and adult cardiac services). Correlations range from −1 to +1, with larger absolute values indicating stronger linear association.

© 2026 Billig JI et al. *JAMA Network Open*.

eMethods 2. Construction of the Effectiveness-of-Care Index Based on HEDIS Measures

eTable 2 shows a component-wise breakdown of our 3 HEDIS measures (i.e. statin therapy for patients with cardiovascular disease, cardiac rehabilitation, and controlling high blood pressure) from 2022-2024. For each individual measure, we assign scores based on its quartile rankings: plans in the first quartile receive a score of 0, those in the second or third quartile receive a score of 0.5, and those in the fourth quartile receive a score of 1.

eTable 2. Component-Wise Breakdown of Cardiac HEDIS Measures Within Medicare Advantage Reduced Cost-Sharing Plans for Cardiologists vs Other, 2022-2024

**Statin Adherence**						
	**2022**		**2023**		**2024**	
**Quality of Plan**	**Cardiology Count (%)**	**Other Count (%)**	**Cardiology Count (%)**	**Other Count (%)**	**Cardiology Count (%)**	**Other Count (%)**
Low (0)	29 (21.7)	1441 (22.8)	19 (13.2)	1282 (19.0)	8 (5.0)	713 (10.7)
Moderate (0.5)	70 (52.2)	2911 (46.1)	76 (52.8)	3093 (45.7)	63 (39.9)	2758 (41.5)
High (1)	33 (24.6)	1459 (23.1)	49 (34.0)	1924 (28.4)	85 (53.8)	2778 (41.8)
Unmatched	2 (1.5)	503 (8.0)	0	470 (6.9)	2 (1.3)	401 (6.0)
**Cardiac Rehabilitation**						
	**2022**		**2023**		**2024**	
**Quality of Plan**	**Cardiology Count (%)**	**Other Count (%)**	**Cardiology Count (%)**	**Other Count (%)**	**Cardiology Count (%)**	**Other Count (%)**
Low (0)	20 (14.9)	1449 (22.9)	8 (5.6)	1147 (17.0)	5 (3.2)	1087 (16.4)
Moderate (0.5)	65 (48.5)	2821 (44.7)	46 (31.9)	2472 (36.5)	34 (21.5)	2056 (30.9)
High (1)	47 (35.1)	1541 (24.4)	90 (62.5)	2680 (39.6)	117 (74.0)	3106 (46.7)
Unmatched	2 (1.5)	503 (8.0)	0	470 (6.9)	2 (1.3)	401 (6.0)
**Controlling Blood Pressure**						
	**2022**		**2023**		**2024**	
**Quality of Plan**	**Cardiology Count (%)**	**Other Count (%)**	**Cardiology Count (%)**	**Other Count (%)**	**Cardiology Count (%)**	**Other Count (%)**
Low (0)	21 (15.7)	1462 (23.1)	7 (4.9)	1035 (15.3)	0	475 (7.1)
Moderate (0.5)	87 (64.9)	2884 (45.7)	51 (35.4)	2705 (40.0)	51 (32.3)	3029 (45.6)
High (1)	24 (17.9)	1465 (23.2)	86 (59.7)	2559 (37.8)	105 (66.4)	2745 (41.3)
Unmatched	2 (1.5)	503 (8.0)	0	470 (6.9)	2 (1.3)	401 (6.0)

HEDIS Measure codes for quality of care included in the index: SPC: Statin Therapy for Patients with Cardiovascular Disease, CRE: Cardiac Rehabilitation, and CBP: Controlling High Blood Pressure.
HEDIS = Healthcare Effectiveness Data and Information Set.

© 2026 Billig JI et al. *JAMA Network Open*.

eTable 3. Quality of Care: Medicare Advantage Reduced Cost-Sharing Plans for Cardiologists vs Other, 2022-2024

	2022			2023			2024		
**Quality of Plan**	**Cardiology Count (%)**	**Other Count (%)**	**P-value[a]**	**Cardiology Count (%)**	**Other Count (%)**	**P-value[a]**	**Cardiology Count (%)**	**Other Count (%)**	**P-value[a]**
**Total**	134	6314		144	6769		158	6650	
Low (0, 0.5, 1)	26 (19.4)	2021 (32.0)	0.047	7 (4.9)	1561 (23.1)	<0.001	0 (0)	747 (11.2)	<0.001
Moderate (1.5, 2)	80 (59.7)	2960 (46.9)		53 (36.8)	2390 (35.3)		38 (24.0)	2523 (38.0)	
High (2.5, 3)	26 (19.4)	830 (13.1)		84 (58.3)	2348 (34.7)		118 (74.7)	2979 (44.8)	
Unknown	2 (1.5)	503 (8.0)		0	470 (6.9)		2 (1.3)	401 (6.0)	

Sensitivity analysis with scoring approach in which each measure was categorized into tertiles and assigned scores of 0, 0.5, and 1 for the first, second, and third tertiles, respectively.
HEDIS Measure codes for quality of care included in the index: SPC: Statin Therapy for Patients with Cardiovascular Disease, CRE: Cardiac Rehabilitation, and CBP: Controlling High Blood Pressure (see appendix table 2 for compositional breakdown).
a. P-values are comparing high-quality plans verses non-high-quality plans (low and medium).
HEDIS = Healthcare Effectiveness Data and Information Set.

© 2026 Billig JI et al. *JAMA Network Open*.

eTable 4. Quality of Care: Medicare Advantage Reduced Cost-Sharing Plans for Cardiologists vs Other, 2022-2024

	2022			2023			2024		
**Quality of Plan**	**Cardiology Count (%)**	**Other Count (%)**	**P-value[a]**	**Cardiology Count (%)**	**Other Count (%)**	**P-value[a]**	**Cardiology Count (%)**	**Other Count (%)**	**P-value[a]**
**Total**	134	6314		144	6769		158	6650	
Low (0, 0.5)	42 (31.3)	1837 (29.1)	0.075	12 (8.3)	1509 (22.3)	<0.001	7 (4.4)	875 (13.2)	<0.001
Moderate (1)	42 (31.3)	2179 (34.5)		34 (23.6)	1832 (27.1)		22 (14.0)	1683 (25.3)	
High (1.5, 2)	48 (35.9)	1795 (28.4)		98 (68.1)	2958 (43.7)		127 (80.3)	3689 (55.5)	
Unknown	2 (1.5)	503 (8.0)		0	470 (6.9)		2 (1.3)	403 (6.0)	

Sensitivity Analysis with only two HEDIS measures included. SPC: Statin Therapy for Patients with Cardiovascular Disease and CBP: Controlling High Blood Pressure (see appendix table 2 for compositional breakdown).
a. P-values are comparing high-quality plans verses non-high-quality plans (low and medium).
HEDIS = Healthcare Effectiveness Data and Information Set.

© 2026 Billig JI et al. *JAMA Network Open*.

**eTable 5.** Component-Wise Breakdown of Non–Cardiac-Related HEDIS Measures Within Medicare Advantage Reduced Cost-Sharing Plans for Cardiologists vs Other, 2022-2024						
**Adults' Access to Preventive/Ambulatory Services**						
	**2022**		**2023**		**2024**	
**Quality of Plan**	**Cardiology Count (%)**	**Other Count (%)**	**Cardiology Count (%)**	**Other Count (%)**	**Cardiology Count (%)**	**Other Count (%)**
Low (0)	18 (13.4)	1441 (22.9)	23 (16.0)	1473 (21.8)	16 (10.1)	1129 (17.0)
Moderate (0.5)	66 (49.3)	2916 (46.2)	61 (42.3)	3346 (49.4)	85 (53.8)	3336 (50.2)
High (1.0)	48 (35.8)	1488 (23.5)	60 (41.7)	1522 (22.5)	55 (34.8)	1841 (27.7)
Unmatched	2 (1.5)	466 (7.4)	0	428 (6.3)	2 (1.3)	344 (5.1)
**Kidney Health Evaluation for Patients with Diabetes**						
	**2022**		**2023**		**2024**	
**Quality of Plan**	**Cardiology Count (%)**	**Other Count (%)**	**Cardiology Count (%)**	**Other Count (%)**	**Cardiology Count (%)**	**Other Count (%)**
Low (0)	21 (15.7)	1457 (23.1)	10 (7.0)	1055 (15.6)	4 (2.5)	345 (5.2)
Moderate (0.5)	84 (62.7)	2922 (46.3)	91 (63.2)	3361 (49.7)	70 (44.3)	3178 (47.8)
High (1)	27 (20.1)	1469 (23.3)	43 (29.8)	1925 (28.4)	82 (51.9)	2783 (41.9)
Unmatched	2 (1.5)	466 (7.4)	0	428 (6.3)	2 (1.3)	344 (5.2)
**Transitions of Care**						
	**2022**		**2023**		**2024**	
**Quality of Plan**	**Cardiology Count (%)**	**Other Count (%)**	**Cardiology Count (%)**	**Other Count (%)**	**Cardiology Count (%)**	**Other Count (%)**
Low (0)	53 (39.5)	1200 (19.0)	8 (5.6)	197 (2.9)	0	190 (2.8)
Moderate (0.5)	41 (30.6)	3181 (50.4)	90 (62.5)	3821 (56.5)	125 (79.1)	2732 (41.1)
High (1.0)	38 (28.4)	1467 (23.2)	46 (31.9)	2323 (34.3)	31 (19.6)	3384 (50.9)
Unmatched	2 (1.5)	466 (7.4)	0	428 (6.3)	2 (1.3)	344 (5.2)

HEDIS Measure codes for quality of care included in the index: AAP: Adults' Access to Preventive/Ambulatory Health Services, KED: Kidney Health Evaluation for Patients with Diabetes, and TRC: Transitions of Care.
HEDIS = Healthcare Effectiveness Data and Information Set.

© 2026 Billig JI et al. *JAMA Network Open*.

eFigure 2. Geographic Variation in County-Level Cardiovascular Rehabilitation Facilities, 2023

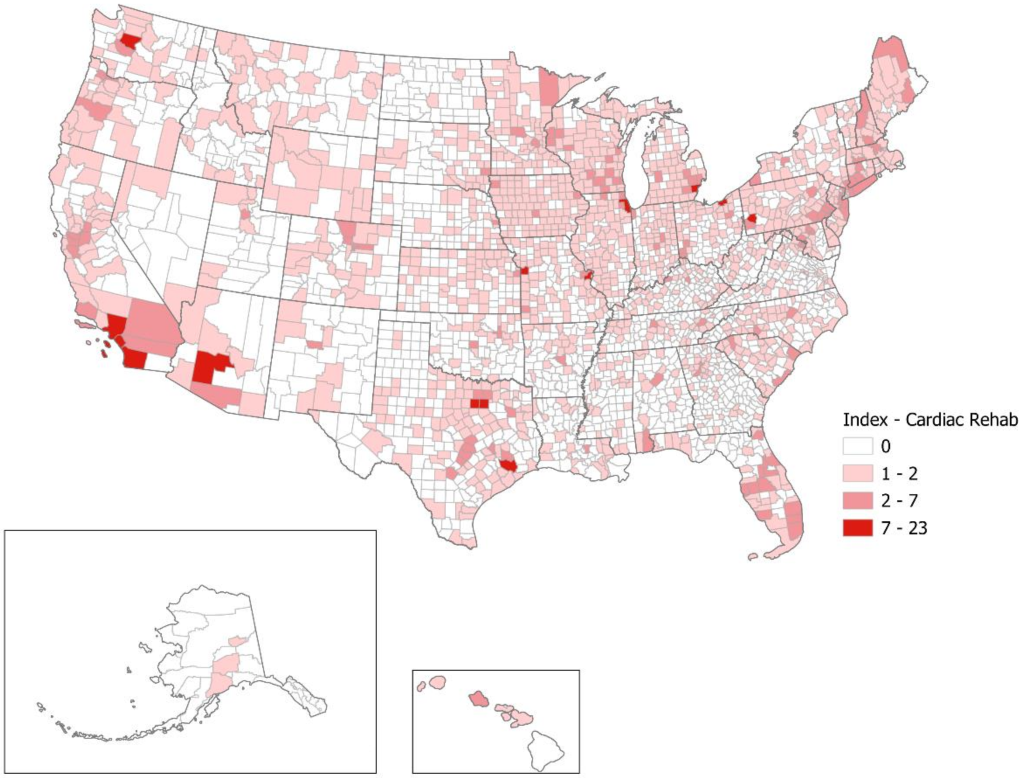

This map illustrates the county-level geographic distribution of cardiac rehabilitation facilities. Map limited to 50 U.S. states and Washington, DC. Zero refers to counties with no cardiac rehabilitation facilities.

© 2026 Billig JI et al. *JAMA Network Open*.

eFigure 3. Geographic Variation in County-Level Adult Cardiology Services, 2023

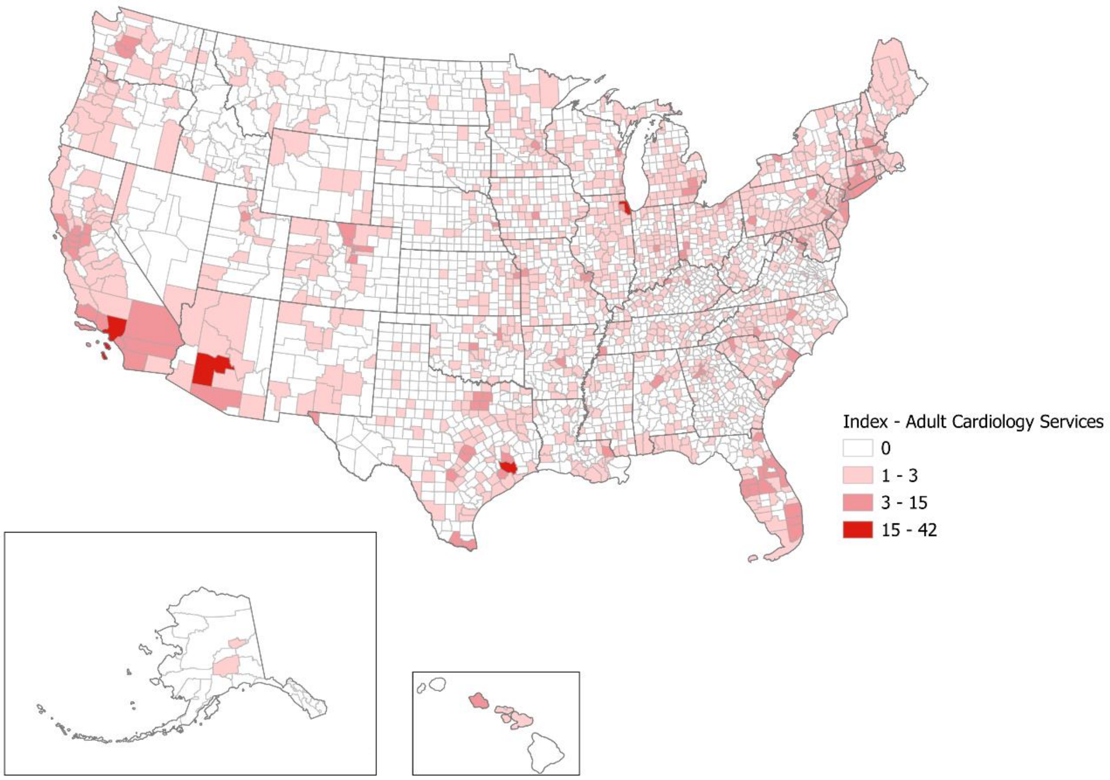

This map illustrates the county-level geographic distribution of adult cardiology services. Map limited to 50 U.S. states and Washington, DC. Zero refers to counties with no adult cardiology services.

© 2026 Billig JI et al. *JAMA Network Open*.

eFigure 4. Geographic Variation in County-Level Cardiovascular ICUs, 2023

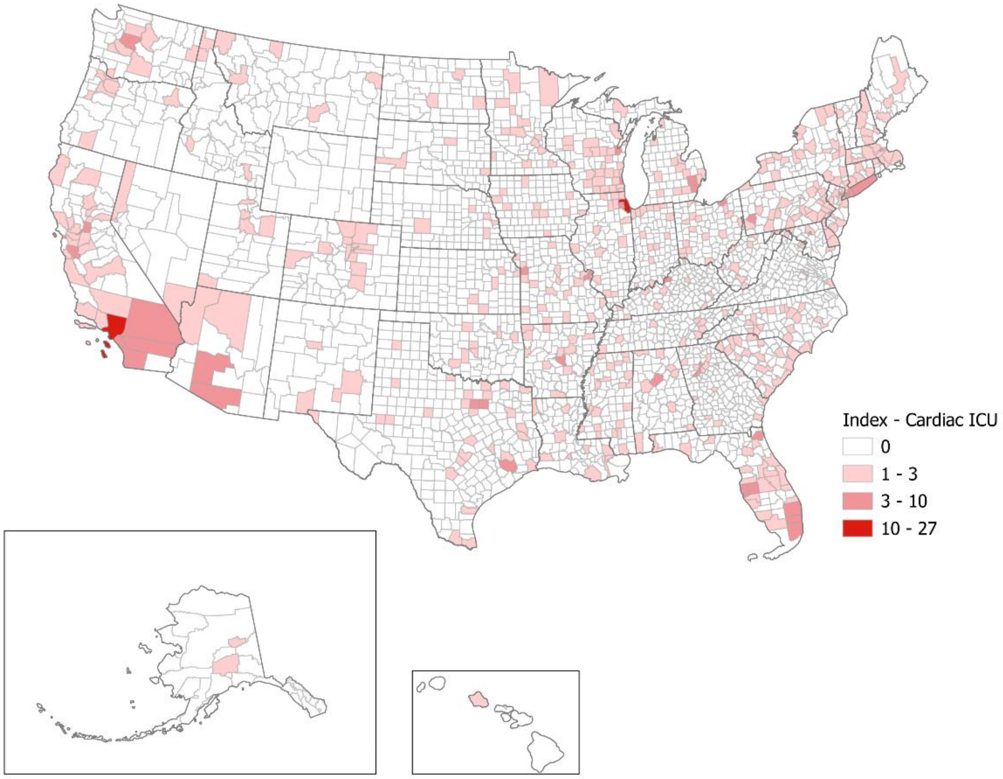

This map illustrates the county-level geographic distribution of cardiac ICUs. Map limited to 50 U.S. states and Washington, DC. Zero refers to counties with no cardiac ICUs.

© 2026 Billig JI et al. *JAMA Network Open*.
