# Supplementary material for: Medicare Advantage Benefits Design and Access to Cardiovascular Care
Source: JAMA Netw Open. 2026 Apr 7;9(4):e265439. doi: 10.1001/jamanetworkopen.2026.5439 (PMC13058761; doi:10.1001/jamanetworkopen.2026.5439)
Supplement: Supplement 2. — Data Sharing Statement [file jamanetwopen-e265439-s002.pdf]

Data Sharing Statement

Billig. Medicare Advantage Benefits Design and Access to Cardiovascular Care. *JAMA Netw Open*. Published April 07, 2026. doi:10.1001/jamanetworkopen.2026.5439

Data
Data available: Yes
Data types: Other (please specify)
Additional Information: Data is publicly available online at: https://www.cms.gov/data-research/statistics-trends-and-reports/medicare-advantagepart-d-contract-and-enrollment-data/benefits-data
How to access data: Data is publicly available online at: https://www.cms.gov/data-research/statistics-trends-and-reports/medicare-advantagepart-d-contract-and-enrollment-data/benefits-data
When available: beginning date: 01-01-2022

Supporting Documents
Document types: None

Additional Information
Who can access the data: Data is publicly available online at: https://www.cms.gov/data-research/statistics-trends-and-reports/medicare-advantagepart-d-contract-and-enrollment-data/benefits-data
Types of analyses: Data is publicly available online at: https://www.cms.gov/data-research/statistics-trends-and-reports/medicare-advantagepart-d-contract-and-enrollment-data/benefits-data
Mechanisms of data availability: Data is publicly available online at: https://www.cms.gov/data-research/statistics-trends-and-reports/medicare-advantagepart-d-contract-and-enrollment-data/benefits-data
